# Disgust and the Politics of Sex: Exposure to a Disgusting Odorant Increases Politically Conservative Views on Sex and Decreases Support for Gay Marriage

**DOI:** 10.1371/journal.pone.0095572

**Published:** 2014-05-05

**Authors:** Thomas G. Adams, Patrick A. Stewart, John C. Blanchar

**Affiliations:** 1 Department of Psychological Science, University of Arkansas, Fayetteville, Arkansas, United States of America; 2 Department of Political Science, University of Arkansas, Fayetteville, Arkansas, United States of America; University of Queensland, Australia

## Abstract

Disgust has been implicated as a potential causal agent underlying socio-political attitudes and behaviors. Several recent studies have suggested that pathogen disgust may be a causal mechanism underlying social conservatism. However, the specificity of this effect is still in question. The present study tested the effects of disgust on a range of policy preferences to clarify whether disgust is generally implicated in political conservatism across public policy attitudes or is uniquely related to specific content domains. Self-reported socio-political attitudes were compared between participants in two experimental conditions: 1) an odorless control condition, and 2) a disgusting odor condition. In keeping with previous research, the present study showed that exposure to a disgusting odor increased endorsement of socially conservative attitudes related to sexuality. In particular, there was a strong and consistent link between induced disgust and less support for gay marriage.

## Introduction

Disgust is increasingly seen as playing an important role in the formation and maintenance of political and social attitudes [1]. Disgust sensitivity and reactivity have been shown to relate to political conservatism generally [2], [3], [3]–[7] and have been implicated in negative and hostile attitudes towards a range of social out-groups, particularly homosexuals [2], [6]–[10]. In keeping with the aforementioned research, some have suggested that disgust is specifically related to conservative attitudes that pertain to social or moral purity [11], and, in particular, sexual purity [4], [12], [13]. One potential reason for these relations is that disgust plays an adaptive role in preserving individuals from the threat of infectious disease [14], [15].

It has been posited that the Behavioral Immune System, which uses the physiological response of disgust as its first line of behavioral defense, shapes political attitudes and resultant policy preferences [5], [16]. As stated by Terrizzi, Shook and McDaniel in their meta-analysis “(A)cross 24 studies, the findings suggest that there is a moderate, positive relationship between behavioral immune system strength and social conservatism” [7]. Despite connections being made between disgust and political ideology, additional work is needed to clarify if disgust sensitivity and/or disgust reactivity are associated with political ideology and what domains of political ideology are related to disgust. Smith and colleagues [2] recently addressed these questions by investigating the relations between disgust sensitivity, disgust reactivity – meaning *in vivo* physiological reaction to disgusting stimuli – and responses to a range of specific political issue items using the well-known Wilson-Patterson scale [17]. Heightened disgust sensitivity and reactivity were both negatively related to attitudes towards gay marriage and premarital sex but not significantly related to a range of other political issues (e.g., gun rights, school prayer, immigration). They also showed that, after controlling for demographic and experimental factors, greater disgust sensitivity was associated with less support for abortion. Said otherwise, this study showed that both trait and state disgust predicted less support for gay marriage and premarital sex and trait disgust predicted less support for abortion. It is noteworthy that, contrary to their predictions, Smith and colleagues failed to show any significant relation between state or trait disgust and attitudes about pornography. This is, however, not too surprising given the limited body of research showing a link between disgust and attitudes about pornography [18].

Evidence from Smith et al. [2] and similar research [9] is compelling but causal interpretations are not possible given their utilization of correlational designs. This limitation was rectified by Inbar and colleagues [3] who experimentally tested the effects of odor induced disgust on self-reported warmth toward homosexuals. They found that, relative to participants in a no odor control condition, participants in the disgust odorant condition reported less warmth toward homosexuals. These findings are similar to those reported by Terrizzi and colleagues [6], who showed that the induction of disgust caused conservative participants to report a decreased willingness to come into contact with homosexuals. What remains to be seen is if the experimental induction of disgust causes attitudinal shifts for other issues related to sexual purity (e.g., premarital sex, abortion, etc.) and if induced disgust causes changes in attitudes towards public policies related to gay marriage.

In the present study we extend previous research by carrying out a true experiment to test the role of induced disgust on the same socio-political attitudes measured by Smith and colleagues. This allows us to test the causal role of disgust on a variety of public policy stances while still focusing on issues of sexual purity, particularly gay marriage. Core/contamination disgust was induced via noxious odorant, which is a primal approach closely reflecting the evolutionary adaptation of this emotion [19]. Moreover, manipulating physical disgust via smell allowed for a continuous induction of the emotion, rather than a brief priming of the emotion prior to measurement. In keeping with the findings of Smith and colleagues [2], we expected the foul disgust odorant, in this case butyric acid, which smells like vomit, to robustly engage the emotion of disgust and decrease approval of gay marriage. Smith and colleagues [2] reported weak effects between state disgust and attitudes about premarital sex and also found non significant relations between state disgust and attitudes about abortion or pornography. Similarly, both Smith and colleagues [2] and Inbar and colleagues [9] reported weak, albeit significant, relations between disgust sensitivity and attitudes about abortion. We therefore expected weak, or perhaps non-significant, effects of our experimental manipulation on attitudes about premarital sex, pornography, and abortion.

## Methods

### Procedures

All procedures were approved by the University of Arkansas Institutional Review Board. All participants provided written informed consent prior to participating in any experimental procedures. Data for the present study can be found at http://dx.doi.org/10.6084/m9.figshare.930730. Community members and students were recruited via an online advertisement and compensated $10 for their time. All procedures were completed in a small group format (*n* = 4–8). Following informed consent, participants were then escorted into a small room (10′x10′x17.5′) with individual desks. An experimenter operated a timed slideshow that displayed experimental instructions while monitoring participants at the back of the room, out of their sight. Participants were assigned to either a disgust odor condition or a control (no odor) condition. Ten minutes prior to participant arrival, an experimenter placed four drops of 99% butyric acid [20], [21] on two small cotton pads and hid these pads at the front and back of the room. Pads were replaced and the room aerated between experimental groups. Immediately upon entering the experimental room, all participants completed disgust ratings and socio-political opinion questions. The Three Domains of Disgust Scale (TDDS), Conservative Liberal Scale, and demographics were completed in a separate, odor-neutral corridor (measures are listed in order of administration below). The disgust induction was not, therefore, expected to have any effect on the TDDS or Conservative Liberal Scale scores.

### Participants

Smith and colleagues reported medium (*r* = .37) to large (*r* = .45) relations between disgust reactivity (state disgust) and attitudes about gay marriage [2]. Power analyses were conducted using G*Power [22]. Results suggest that, given alpha of .05 and power of .80, a sample of 33 total participants would be required to detect an effect size of .45, and a sample of 54 total participants would be required to detect an effect size of .37. Smith and colleagues reported small relations (*r*s = .10 to .28) between disgust reactivity and attitudes about other issues of sexual purity (e.g., premarital sex, pornography, abortion). Given an alpha of .05 and power of .80, a sample of 779 total participants would be required to detect an effect size of .10 and a sample of 95 total participants would be required to detect an effect size of .28. A total of 57 participants were included in the present study, thirty of whom were randomly allotted to the control group. As such, the present sample size is only sufficiently powered for medium to large effects.

A majority of participants were Caucasian/white (*n* = 51), self-described Christian (*n* = 40), and heterosexual (*n* = 53); there were no significant differences between the two experimental groups on any of the aforementioned demographic variables. There was a significant difference in gender distribution between the two experimental groups, *X^2^* (1)  = 6.22, *p* = .01, with relatively more female than male participants in the control group (*n* = 21 and 9, respectively) compared to the disgust odor group (*n* = 10 and 17, respectively). The average age of the sample was 32.37 (*SD* = 14.15). The control group (*M* = 35.33, *SD* = 15.59) was marginally older than the disgust odor group (*M* = 29.07, *SD* = 11.79), *F* (1, 55)  = 2.87, *p* = .10.

### Measures

#### Disgust ratings

As a manipulation check, participants were asked to select a number ranging from 0 ‘Not at all’ to 8 ‘Very much so’ to indicate the degree to which they currently felt “disgusted, nauseated, repulsed.”

#### Socio-political opinion questions

The Wilson-Patterson short-response format [17], which asks participants if they agree, disagree, or are uncertain on a range of currently salient public policy issues, was used to measure socio-political attitudes. Responses were recoded so that 1 =  agree, 2 =  uncertain, and 3 =  disagree. Participants responded to 16 items concerning social, economic, regulatory, and international public policy. Even though our predictions were only concerned with sex-related attitudes, we opted to include other Wilson-Patterson items to allow for a direct comparison between our findings and those published by Smith and colleagues [2]. Eight items were worded such that higher scores reflected more agreement with conservative attitudes and eight items were worded such that lower scores reflected less agreement with conservative attitudes (see Table 1). Participants also completed four 5-point items that were specifically concerned with same sex marriage. The first of the aforementioned items was scaled from 1 ‘strongly oppose’ to 5 ‘strongly favor’ and was the same item used by Smith and colleagues [2] while the latter three items, which were developed for the present study, were scaled from 1 ‘strongly agree’ to 5 ‘strongly disagree’ (Table 1).

**Table 1:** Descriptive data and summary of ANOVAs split by experimental groups.

Dependent variables	Condition	Mean (SD)	With Covariates *F*(ηp^2^)	Without Covariates *F* (ηp^2^)
***5-Pt. Gay Marriage Items***				
The federal government should support a constitutional amendment banning gay marriages*	Control	1.57 (1.04)	7.80 (.13)^b^	4.36 (.07)^a^
	Odor	2.33 (1.69)		
I should be allowed to marry whomever I want to, even if it is a member of the same sex	Control	1.70 (1.26)	15.42 (.23)^c^	7.38 (.12)^b^
	Odor	2.78 (1.72)		
If a close friend or family member were gay, I would support their right to having a same-sex marriage.	Control	1.53 (1.01)	17.29 (.25)^c^	9.70 (.15)^b^
	Odor	2.67 (1.69)		
Same sex marriage should be legalized nationwide	Control	1.80 (1.38)	13.88 (.21)^c^	5.49 (.09)^a^
	Odor	2.74 (1.65)		
***Wilson-Patterson Items***				
Gay marriage	Control	1.37 (.67)	12.54 (.19)^c^	4.75 (.08)^a^
	Odor	1.81 (.88)		
Premarital sex	Control	1.33 (.66)	6.50 (.11)^a^	2.95 (.05)
	Odor	1.71 (.95)		
Pornography	Control	1.83 (.87)	6.94 (.12)^b^	1.48 (.03)
	Odor	2.11 (.85)		
Abortion rights	Control	1.50 (.86)	1.47 (.03)	0.31 (.01)
	Odor	1.63 (.88)		
School prayer*	Control	2.30 (.92)	2.68 (.05)	1.40 (.03)
	Odor	2.00 (1.0)		
Illegal immigration	Control	2.30 (.88)	0.24 (.01)	0.78 (.01)
	Odor	2.48 (.64)		
Death penalty	Control	1.93 (.91)	0.00 (.00)	0.19 (.00)
	Odor	2.04 (.90)		
Biblical truth	Control	2.20 (.81)	11.24 (.18)^c^	6.04 (.10)^a^
	Odor	1.67 (.83)		
Welfare spending	Control	1.67 (.84)	0.95 (.02)	0.11 (.00)
	Odor	1.74 (.81)		
Tax cuts*	Control	1.73 (.74)	0.26 (.01)	0.11 (.00)
	Odor	1.67 (.78)		
Gun control	Control	1.73 (.86)	0.62 (.01)	0.43 (.01)
	Odor	1.89 (.93)		
Military spending*	Control	2.03 (.85)	0.61 (.01)	0.10 (.00)
	Odor	1.96 (.85)		
Warrantless searches*	Control	2.77 (.57)	.054 (.01)	1.77 (.03)
	Odor	2.93 (.27)		
Small government*	Control	1.77 (.77)	0.06 (.00)	0.02 (.00)
	Odor	1.74 (.66)		
Foreign aid	Control	1.70 (.84)	2.01 (.04)	0.65 (.01)
	Odor	1.89 (.93)		
Free trade	Control	1.43 (.73)	0.10 (.00)	0.08 (.00)
	Odor	1.48 (.58)		

Note. All ANCOVA *df = *1, 52 and ANOVA *df* = 1, 56. “*” denotes items for which agreement (i.e., lower score) are consistent with a more conservative stance. Covariates included for ANCOVA analyses were gender, age, and moral disgust propensity.

Superscript “^a^” denotes *p*≤.05, superscript “^b^” denotes *p*≤.01, superscript ”^c^” denoted *p*≤.001.

#### Conservatism Liberal Scale

The Conservatism Liberal Scale [23] is a one-item measure presenting the following: “We hear a lot of talk these days about liberals and conservatives. Here is a 7-point scale on which the political views that people might hold are arranged from extremely liberal to extremely conservative.” Participants are then asked to place themselves on the scale from 1 (extremely conservative) to 7 (extremely liberal).

#### Three Domains of Disgust Scale

The TDDS [13] is a 21-item self-report scale that measures three distinct domains of disgust sensitivity (moral, sexual, and pathogen). Each factor is represented by seven items measured on a zero to 6-point scale, ranging from ‘not at all disgusting’ to ‘extremely disgusting’. The TDDS has strong psychometric properties [24] and internal consistencies of the TDDS factors were moderate within the present sample, (α_Moral Disgust_  = 0.83), (α_Sexual Disgust_  = 0.82), and (α_Pathogen Disgust_  = 0.79).

## Results

A series of analysis of variance (ANOVA) tested dispositional differences between experimental groups on political ideology (conservative-liberal) and moral, sexual, and pathogen sensitivities. Results revealed that the no odor control group endorsed significantly more moral disgust sensitivity (*M* = 31.14, *SD* = 5.90) than the disgust odor group, (*M* = 27.30, *SD* = 8.23), *F* (1, 55)  = 4.16, *p* = .05. The two experimental groups did not differ in terms of political ideology, sexual disgust, or pathogen disgust, (all *ps* >.10). The TDDS was completed several minutes after the disgust induction and in a corridor that was separate from the disgust odor infused room. If the disgust induction did have a lingering effect on TDDS scores, then participants would have likely also scored higher on the pathogen disgust subscale, which was not the case. It is, therefore, unlikely that the between group difference in moral disgust was due to the disgust induction.

An analysis of variance (ANOVA) showed that participants in the disgust odor condition reported more subjective disgust during the manipulation check (*M* = 3.85, *SD* = 2.03) than participants in the control condition (*M* = 0.37, *SD* = 1.03), *F* (1, 52)  = 68.56, *p*<.01, *d* = 2.16. This effect remained significant after adding gender, age, and moral disgust sensitivity as covariates (see Participants section and the previous paragraph), *F* (1, 52)  = 48.28 *p*<.01.

To account for between group differences in demographic and dispositional variables, a series of ANCOVAs – with gender, age, and moral disgust sensitivity as covariates – were carried out with the 4 sexual purity Wilson-Patterson and four 5-point gay marriage items. False discovery rates were controlled for using adaptive alpha correction procedures outlined by Benjamini and Hoochberg [25]. As expected, attitudes about gay marriage were affected by the disgust induction, with participants in the disgust odor condition reporting less agreement with gay marriage (as measured by the one Wilson-Patterson item and three 5-point items) and less opposition (and more support) to a constitutional ban on gay marriage (*p*s<.01). Also consistent with our predictions and the findings of Smith et al. [2], we found that participants in the disgust odor condition disagreed significantly more with the Wilson-Patterson premarital sex (*p*≤.01) item. Consistent with our own prediction but in contrast with the findings of Smith and colleagues [2], participants in the disgust odor condition reported more disagreement with pornography (*p*≤.01). While participants in the disgust odor condition did report less agreement with abortion rights, this effect was not significant (*p* = .23). Exploratory analyses revealed that participants in the disgust odor condition were in significantly more agreement with Biblical truth (*p*<.001). The experimental groups did not significantly differ on any of the remaining Wilson-Patterson items (all *p*s>.05). See Table 1 for a summary.

Group differences on all items were also analyzed with a series of ANOVAs. Consistent with ANCOVA results, univariate ANOVAs showed that participants in the disgust odor condition reported significantly less support for allowing individuals to marry whomever they wish (*p* = .009) and for allowing friends or family members to pursue same sex marriages (*p* = .003). Participants in the disgust odor condition also reported less agreement with the Wilson-Patterson gay marriage item (*p* = .034), less agreement with nationwide legalization of same-sex marriage (*p* = .023), and less opposition to an amendment banning gay marriage (*p* = .041); however, these effects were not significant after alpha correction. While participants in the disgust odor condition reported less agreement with premarital sex, pornography, and abortion, these effects were not statistically significant (all *p*s>.05). Consistent with ANCOVA results, univariate analyses showed that participants in the disgust odor condition reported more support for the Biblical truth item (*p* = .017). Scores on the remaining Wilson-Patterson items did not significantly differ between the two experimental groups (all *p*s>.10). See Table 1 for details.

Given that several effects were enhanced by the inclusion of covariates, correlations between age, moral disgust, and items measuring attitudes about sexual purity were inspected. There were strong correlations between age and several other variables, with older participants reporting more moral disgust sensitivity (*r* = .43, *p*≤.01), less agreement with legalizing gay marriage (*r* = .35, *p*≤.01), and less agreement with the Wilson-Patterson gay marriage item (*r* = .34, *p*≤.05). Similarly, participants who reported more moral disgust sensitivity also reported more conservative attitudes for all items related to gay marriage (*r*s≤.30 and *p*s≤.05). A series of ANOVAs also revealed that female participants disagreed more with pornography compared with male participants. A two-way ANOVA with gender and experimental condition as the IVs and attitudes about pornography as the DV failed to show a significant gender by condition interaction *F* (1, 53)  = 0.83, *p* = .37. None of the covariates were significantly related to attitudes about premarital sex (*p*s>.05). Taken as a whole, inspection of the relations between covariates and the dependent variables suggests that gender only affected attitudes about pornography, age and moral disgust sensitivity affected attitudes related to gay marriage, and no covariates were significantly related to attitudes about premarital sex.

**Figure 1:**
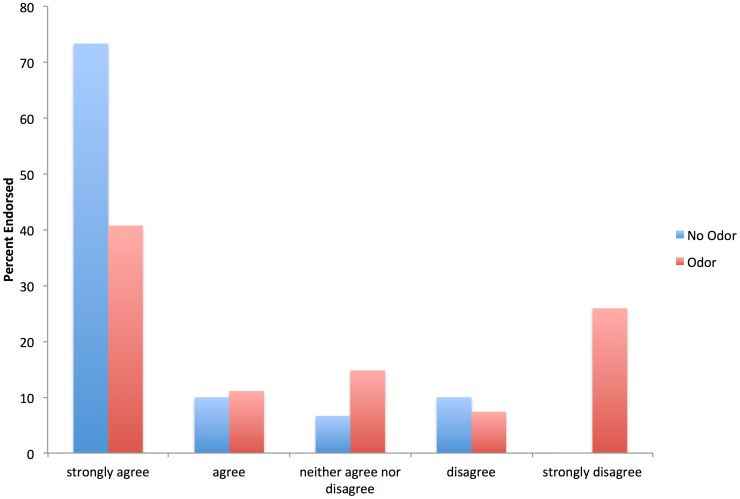
Attitudes about the following statement: “If a close friend of family member were gay, I would support their right to having a same-sex marriage.”

Visual inspection of the distribution of the friend of family member same-sex marriage item – which demonstrated the largest effect size (*d* = .82) – suggested that the disgust odor induction caused attitudes to literally shift to the right (Figure 1). A smaller percentage of participants in the disgust odor condition strongly agreed with allowing friends or family members to pursue same-sex marriage and a larger percentage in this group strongly disagreed with such an idea. For example, zero participants in the no odor control group compared to 25.9% of participants in the disgust odor group strongly disagreed with allowing friends or family to pursue same-sex marriage. Interestingly, the experimental manipulation did not appear to dramatically affect the percentage of participants who endorsed more moderate attitudes (i.e., somewhat oppose to somewhat favor).

## Discussion

Disgust has an important, causal relationship with political attitudes concerning sexual conduct. In an experiment manipulating odor-based disgust, participants exposed to the smell of butyric acid reported increased subjective disgust and more politically conservative attitudes concerning gay marriage, premarital sex, pornography, and Biblical truth. Rejection of gay marriage was a particularly strong response to the disgust-inducing odor, perhaps because of the connection between homosexuality and perceptions of sexual impurity [4], [26]. The finding that belief in Biblical truth was greater among participants in the disgust odor condition was unexpected but is nonetheless consistent with previous work showing a relation between disgust and scrupulosity or being careful to avoid doing wrong. Indeed, Olatunji presented data that suggested a causal chain between disgust, scrupulosity, and conservative beliefs about homosexuality [10]. Future research should continue testing the causal effects of disgust on religious beliefs and the effects of religion on sexual attitudes.

We report non-significant findings for a variety of non-sexual socio-political attitudes (e.g., tax cuts, illegal immigration, etc.), but we do not interpret these null findings as evidencing an absence of relations between disgust and non-sexual socio-political attitudes. Replication with larger samples is needed before unconditional conclusions can be drawn, particularly with regard to smaller effects, which the present study was not appropriately powered to detect [e.g., abortion (see Participants section)]. Nonetheless, the present findings suggest that odor-induced disgust had a stronger effect on sexual attitudes than non-sexual attitudes (save for Biblical truth). The effects of disgust on sex-related attitudes, particularly those concerning gay marriage, are especially convincing when the present results are considered in the context of previous research [2], [4], [9]. For example, Smith and colleagues [2] reported very similar findings: greater changes in skin conductance following disgust provocation predicted stronger conservative views on gay marriage and premarital sex. However, the experimental approach of our study advances causal claims, particularly with regard to the effects of state disgust on views related to individual liberties pertaining to sexual orientation.

Participants in the no odor control condition were older, endorsed more moral disgust sensitivity, and were comprised of more females when compared to participants in the disgust odor condition. Gender was related to decreased agreement with pornography while age and moral disgust sensitivity were related to decreased agreement and support for same-sex marriage. It is, therefore, understandable that the inclusion of age, gender, and moral disgust sensitivity as covariates enhanced the magnitude of the experimental effect on attitudes about pornography and gay marriage. These results also suggest that extra emphasis should be placed on most of the ANCOVA results relative to the univariate analyses. Given the absence of relations between any of the covariates and premarital sex, it is difficult to determine precisely why the addition of covariates boosted this specific effect. The simplest explanation is that the addition of covariates reduced residual variance enough to push up effect size estimates and push down alpha estimates below significance thresholds. Regardless, the present findings, particularly those that were only significant with covariates (i.e., attitudes about premarital sex and pornography) must be interpreted with caution, as covariate analyses do no completely resolve sampling issues [27]. Future research should attempt to replicate the present findings with experimental groups that do not differ on important demographic and individual difference variables.

These data are consistent with theory delineated by Tybur and colleagues [13], which argues that disgust functions to decrease the occurrence (both in the self and society) of sexual behaviors that are perceived as increasing risk of pathogen transmission. Relatedly, and as noted by Haidt and Graham [11], conservative attitudes are driven not only by harm avoidance, but also by concerns about purity. According to these theories, shifts toward politically conservative views on sex may be basic, adaptive, and self-protective responses against perceived spread of pathogens or moral threats. When disgust is evoked, the behavioral immune system engages avoidance to prevent infection (e.g., less interpersonal contact [28]) and appears to moralize sexual conduct in ways that underlie conservative values of purity and sanctity [11], [26]. As seen in the results of our study, it is possible that exposure to a disgusting odorant caused increased feelings of disgust, which in turn activated the harm avoidance system and motivated a desire for purity (cleanliness). Once these two systems were activated, it is possible that participants began to adopt attitudes that they perceived as decreasing social harm and/or increasing moral purity.

There is a growing literature indicating that disgust has important consequences for political views and policy preferences. In the research presented here, exposure to a disgusting odor caused greater endorsement of conservative views, including: rejecting gay marriage, restricting sex to marriage, disapproving of the use of pornography, and increased beliefs in Biblical truth. Odor induced conservative shifts concerning gay marriage were particularly robust. It is possible that some forms of political conservatism, particularly those related to sex and sexuality, are basic and inherent in some populations and can readily emerge under threatening or taxing conditions [4], [29], [30].
